# ID 51 - Talk Show: experiências exitosas da engenharia clínica

**DOI:** 10.5327/2237-9622.2025.v34s1.51

**Published:** 2025-11-25

**Authors:** Fotini Santos Toscas, Alzinete Cunha, Ana Paula Lemes Jesus dos Santos, Léria Rosane Holsbach

## Abstract

**Introdução:**

O Talk Show integrou a programação científica do 2º Seminário Latino-Americano de Engenharia Clínica, realizado durante a Feira Hospitalar, em maio de 2024, em São Paulo. Reconhecida como a principal feira do setor de saúde na América Latina, a Feira Hospitalar consolidou-se ao longo de mais de três décadas como o ponto de encontro por excelência das diversas comunidades da saúde. A edição de 2024 contou com a presença de 80 mil profissionais, 800 palestrantes e visitantes de 80 países, proporcionando oportunidades únicas de networking e troca de conhecimento.

O 2º Seminário Latino-Americano de Engenharia Clínica, organizado pela Associação Brasileira de Engenharia Clínica (Abeclin), reuniu especialistas do Brasil, Peru, Paraguai e Uruguai para discutir os avanços e os desafios da Engenharia Clínica na América Latina e no mundo.

O Talk Show teve como objetivo promover o compartilhamento de experiências bem-sucedidas entre profissionais da Engenharia Clínica, proporcionando um espaço colaborativo para a troca de saberes práticos. Também visou encorajar a demonstração de novas abordagens para solucionar desafios comuns da área. Os trabalhos apresentados abrangeram uma diversidade de temas, com especial destaque para a ATS. Foram aprovados 11 trabalhos submetidos para apresentação oral no Talk Show, sendo quatro com interfaces diretas com estudos de ATS:

– Análise de Custo-Efetividade do Equipamento de Colonoscopia Auxiliado por Inteligência Artificial versus Colonoscopia Convencional na Detecção de Pólipos Adenomatosos e Prevenção do Câncer Colorretal: uma avaliação econômica sob a perspectiva do SUS
– Atualização do Arsenal Tecnológico da Radioterapia no Sistema Único de Saúde
– Dados de Mundo Real em Dispositivos Médicos no Cenário Brasileiro: Estudo de Caso dos Kits Diagnósticos para Covid-19
– Desenvolvimento de Equipamentos Médicos no Brasil: Desafios e Etapas – Estudo de Caso do Desenvolvimento de um Ventilador Pulmonar

Os quatro melhores trabalhos apresentados foram premiados, sendo dois do eixo de ATS: "Atualização do Arsenal Tecnológico da Radioterapia no Sistema Único de Saúde" e "Dados de Mundo Real em Dispositivos Médicos no Cenário Brasileiro: Estudo de Caso dos Kits Diagnósticos para Covid-19".

**Método:**

A expectativa é que, durante o V Congresso da Rebrats, seja promovida a apresentação da iniciativa com breve relato dos trabalhos premiados no eixo de ATS, criando uma oportunidade para dar visibilidade às contribuições dos profissionais de Engenharia Clínica na área ATS. Conforme a publicação da Organização Mundial da Saúde (OMS), engenheiros biomédicos e clínicos estão entre os principais profissionais responsáveis pelo ciclo de vida dos dispositivos médicos, incluindo a Avaliação de Tecnologias em Saúde (ATS). Para isso, será necessário um espaço equipado com sistema de som e projeção.

